# Donor-specific antibodies require preactivated immune system to harm renal transplant

**DOI:** 10.1016/j.ebiom.2016.06.006

**Published:** 2016-06-05

**Authors:** Caner Süsal, Bernd Döhler, Andrea Ruhenstroth, Christian Morath, Antonij Slavcev, Thomas Fehr, Eric Wagner, Bernd Krüger, Margaret Rees, Sanja Balen, Stela Živčić-Ćosić, Douglas J. Norman, Dirk Kuypers, Marie-Paule Emonds, Przemyslaw Pisarski, Claudia Bösmüller, Rolf Weimer, Joannis Mytilineos, Sabine Scherer, Thuong H. Tran, Petra Gombos, Peter Schemmer, Martin Zeier, Gerhard Opelz, A Collaborative Transplant Study Report

**Affiliations:** a Transplantation Immunology, Institute of Immunology, Heidelberg, Germany; b Division of Nephrology, University of Heidelberg, Heidelberg, Germany; c Department of Immunogenetics, Institute for Clinical and Experimental Medicine (IKEM), Prague, Czech Republic; d Division of Nephrology, University Hospital Zurich, Zurich, Switzerland; e Immunology and Histocompatibility Laboratory, CHU de Québec-Université Laval, Québec, QC, Canada; f University Medical Centre Mannheim, Mannheim, Germany; g Welsh Transplantation and Immunogenetics Laboratory, Cardiff, United Kingdom; h Departments of Transfusion Medicine and Nephrology, Dialysis, Kidney Transplantation, University Hospital Centre, Rijeka, Croatia; i Oregon Health and Science University, Portland, OR, USA; j Department of Nephrology and Renal Transplantation, Leuven, Belgium; k Blood Transfusion Center, University Hospitals Leuven, Leuven, Belgium; l Transplantation Surgery, University of Freiburg, Freiburg, Germany; m Department of General and Transplant Surgery, Innsbruck Medical University, Innsbruck, Austria; n Department of Internal Medicine, University Clinic of Giessen and Marburg, Giessen, Germany; o Department of Transplantation Immunology, Institute of Transfusion Medicine, University Clinic Ulm, Ulm, Germany; p Transplantation and General Surgery, University of Heidelberg, Heidelberg, Germany; q Department of Microbiology, Infectious Diseases and Immunology, Université Laval, Québec, QC, Canada

**Keywords:** Single antigen bead, HLA antibodies, Donor-specific antibodies, sCD30, Kidney transplantation, Graft outcome

## Abstract

**Background:**

It is an unresolved issue why some kidney transplant recipients with pretransplant donor-specific HLA antibodies (DSA) show a high transplant failure rate, whereas in other patients DSA do not harm the graft. We investigated whether help from preactivated T-cells might be necessary for DSA to exert a deleterious effect.

**Methods:**

The impact of pretransplant DSA and immune activation marker soluble CD30 (sCD30) on 3-year graft survival was analyzed in 385 presensitized kidney transplant recipients.

**Findings:**

A deleterious influence of pretransplant DSA on graft survival was evident only in patients who were positive for the immune activation marker sCD30. In the absence of sCD30 positivity, 3-year graft survival was virtually identical in patients with or without DSA (83.1 ± 3.9% and 84.3 ± 2.8%, *P* = 0.81). A strikingly lower 3-year graft survival rate of 62.1 ± 6.4% was observed in patients who were both sCD30 and DSA positive (HR 2.92, *P* < 0.001). Even in the presence of strong DSA with ≥ 5000 MFI, the 3-year graft survival rate was high if the recipients were sCD30 negative.

**Interpretation:**

Pretransplant DSA have a significantly deleterious impact on graft survival only in the presence of high pretransplant levels of the activation marker sCD30.

## Highlights

• Pretransplant DSA have a deleterious impact on graft survival only in the presence of high pretransplant serum levels of sCD30.
• The majority of patients with pretransplant DSA might be transplanted safely without special pretreatment measures.

Kidney transplantation in the presence of donor-specific HLA antibodies (DSA) is associated with a high failure rate due to antibody-mediated rejection. Many centers avoid transplantations if DSA are present. Others perform such transplantations after removal of DSA by apheresis under potent immunosuppression.

We provide strong evidence that DSA positive recipients reject their grafts at a high rate only if the immune activation marker sCD30 is also high, suggesting that T-cell help from an activated immune system is necessary for pretransplant DSA to exert a deleterious effect on the graft.

High-risk patients with DSA and sCD30 may benefit from special treatment measures. The presence of DSA alone may not be deleterious.

## 1 Introduction

Kidney transplants involving recipients who possess lymphocytotoxic antibodies against mismatched HLA antigens of the donor are at high risk of antibody-mediated rejection. In today's clinical practice such transplants are generally avoided (Tait et al., 2013). A complement-dependent cytotoxicity (CDC) crossmatch that detects donor-directed antibodies in the patient's sera was introduced in the 1970's and, supplemented by the flow cytometry version, allows the exclusion of unfavorable recipient donor combinations (Patel and Terasaki, 1969). The CDC technique has the drawback of not being highly sensitive and has been criticized for not detecting all clinically relevant antibodies. During recent years, more sensitive solid-phase assays based on ELISA, flow cytometry and Luminex^®^ platforms were introduced for detection and specification of donor-specific HLA antibodies (DSA), and the pretransplant inclusion of HLA antibody specificities in the recipient's waiting list profile for allowing exclusion of ‘unacceptable HLA antigen mismatches' in the ‘virtual crossmatch’ has become routine practice (Tait et al., 2013). Of modern antibody assays, the Luminex^®^ single antigen bead (SAB) technique has, despite the drawback that it occasionally gives false positive results due to the presence of denatured HLA on the bead surface (Cai et al., 2009), the highest sensitivity and resolution and is therefore used widely.

Contradictory results were obtained with respect to whether all pretransplant DSA detected by the sensitive SAB technique are deleterious (van den Berg-Loonen et al., 2008, Eng et al., 2008). In a retrospective study, we found that the incidence of pretransplant DSA was not higher in kidney recipients with graft loss than in recipients with functioning grafts if the antibodies were reactive exclusively in the SAB assay but not in the less sensitive CDC or ELISA assays (Susal et al., 2011). While the antibodies often seemed to act as mediators of allograft destruction, we and others noticed that, in some recipients, antibodies persisted but did no harm to the graft or disappeared (Susal et al., 2015, Knight et al., 2013).

The complement (C)-activating capacity of DSA, indirectly assessed by classical pathway component C1q binding, was proposed as a discriminator between deleterious and non-harmful antibodies (Chen et al., 2011, Loupy et al., 2013). However, in today's practice most patients with pretransplant C1q-binding DSA are excluded from transplantation because these antibodies, if sufficiently strong, react positively in the CDC crossmatch. In retrospective testing, pretransplant C1q-binding DSA were therefore rare in patients who received a transplant after routine crossmatching (Otten et al., 2012). Even if detected pretransplant, they often disappeared after transplantation without inflicting harm (Schaefer et al., 2016). In a small cohort of six patients with graft loss due to antibody-mediated rejection, we obtained preliminary evidence that the deleterious effect of pretransplant DSA might be related to *T*-cell help originating from a preactivated immune system (Schaefer et al., 2016). T-cell support is required for the switch of B cells to plasma cells that produce antibodies with high affinity for mismatched donor HLA. Previous data showed that increased levels of the immune system activation marker soluble CD30 (sCD30) are associated with an increased risk of graft loss (Susal et al., 2002, Heinemann et al., 2007, Saini et al., 2008).

Because of our previous failure to find an association of graft failure with pretransplant DSA that exclusively reacted in the exquisitely sensitive SAB assay (Susal et al., 2011), we focused in the present study on pretransplant sera containing CDC- or ELISA-reactive antibodies, selected without regard to donor-specificity. Antibodies detected with these assays of lower sensitivity have been found in the past to correlate with kidney graft outcome (Schonemann et al., 1998, Susal and Opelz, 2002). In patients possessing such antibodies, we analyzed whether pretransplant HLA antibodies with donor specificity, as identified in the highly sensitive SAB assay, might require a preactivated immune system, as indicated by high serum sCD30 at the time of transplantation, as a prerequisite for unfolding a deleterious action.

## 2 Methods

### 2.1 Patients

Adult (≥ 18 years old) recipients of deceased donor kidney transplants who were transplanted 1996–2011 were studied retrospectively. Patients with multi-organ transplants were excluded. We selected all 385 patients from the Collaborative Transplant Study (CTS) combined serum and DNA study (www.ctstransplant.org) whose last pretransplant serum was reported by the participating centers to be positive in the CDC panel reactivity assay (PRA) or who tested positive in the CTS serum study for ELISA-reactive HLA antibodies (AbScreen, Biorad, Dreieich, Germany). These patients were termed “presensitized” and their frozen-stored serum and DNA specimen were used for additional testing. Based on previous findings, an optical density (OD) of ≥ 0.300 in ELISA and > 0% reactivity in the CDC-PRA assay were used as cut-offs for positivity (Susal and Opelz, 2002). Thirteen transplant centers participated both in the serum and the DNA study, and transplants from these centers were selected for the current project. The availability of DNA on recipients and their respective donors allowed for the retrospective typing of HLA A, B, C, DRB1, DRB3, DRB4, DRB5, DQA1, DQB1, and DPB1 antigens, and thereby the precise definition of DSA. Patient consent and ethics committee approval was obtained and the investigations were performed in accordance with the Declaration of Helsinki. Demographic characteristics of the patients are shown in Table 1. Only 11 (3%) of the patients had an incomplete 3 year follow-up. Characteristics of patients subdivided into further subgroups according to sCD30 positivity or negativity are shown in Supplementary Table S1.

### 2.2 Measurements

The sera of the 385 presensitized patients were tested in the Heidelberg laboratory for serum sCD30 content using the ELISA kit of eBioscience (San Diego, USA) and for HLA antibodies using the LABScreen kits of One Lambda (Canoga Park, CA) which utilize single HLA antigen-coated beads and enable the identification of IgG alloantibody specificities against HLA A, B, C, DRB1, DRB3, DRB4, DRB5, DQA1, DQB1, and DPB1. No clinical cut-off for these assays is recommended by the provider companies. The receiver operating characteristic curve analysis, in which 3-year graft as well as death-censored graft survival was analyzed at five different cut-offs (70, 80, 90, 100, 110 ng/ml), indicated 80 ng/ml as the most suitable cut-off for sCD30 testing. Based on experience from previous studies (Susal et al., 2011), a mean fluorescence intensity (MFI) of ≥ 1000, was considered positive for HLA antibody reactivity. For high resolution typing of HLA A, B, C, DRB1, DQA1, and DQB1 antigens at the allele level, CTS PCR-SSP Tray and CTS-Sequence Kits (Heidelberg, Germany), and for HLA DRB3, DRB4, DRB5 and DPB1 typing Olerup SSP kits (Saltsjöbaden, Sweden) were used. All DSA positive sera were analyzed for the presence of C1q-binding antibodies using the C1qScreenTM kit of One Lambda and applying a cut-off of 300 MFI (Chen et al., 2011).

### 2.3 Statistical analysis

Graft as well as death-censored graft survival rates were computed according to the Kaplan-Meier method and are expressed as % ± standard error. Log-rank test, Fisher's exact test, Mann-Whitney U test and multivariable Cox regression were used for statistical analysis. In multivariable analysis, geographic region, year and number of transplant, recipient and donor gender and age, original disease causing end stage renal failure, pretransplant clinical evaluation of the patient, HLA-A + B + DR mismatches, pretransplant time on dialysis, and intention-to-treat immunosuppressive therapy (antibody induction, calcineurin inhibitors, anti-proliferatives) were considered as confounders. Patients negative in sCD30 and DSA testing served as reference group for the calculation of hazard ratios. *P* values below 0.05 were considered statistically significant. The software package IBM^®^ SPSS^®^ Statistics version 22.0 (SPSS Inc., IBM Corporation, Somers, NY, USA) was used.

### 2.4 Role of the funding source

No outside funding was obtained for this study.

## 3 Results

115 of the 385 (30%) presensitized patients had a pretransplant sCD30 serum content of ≥ 80 ng/ml and were termed sCD30 positive. The 3-year graft survival rate in these 115 recipients was 73.8 ± 4.1%, significantly lower than the 83.8 ± 2.3% rate in the remaining 270 recipients who were presensitized but sCD30 negative (log rank *P* = 0.022). All 385 presensitized patients as determined by CDC or ELISA testing also were positive for HLA antibodies in the highly sensitive SAB assay, and 154 of the 385 (40%) possessed SAB-detected antibodies specific against mismatched donor HLA (= donor-specific antibodies, DSA). The 3-year graft survival in these 154 DSA positive patients was 75.1 ± 3.5%, significantly lower than the 84.7 ± 2.4% rate in the 231 patients who had antibodies that were not directed against donor HLA (*P* = 0.017, data not shown).

**Fig. 1:**
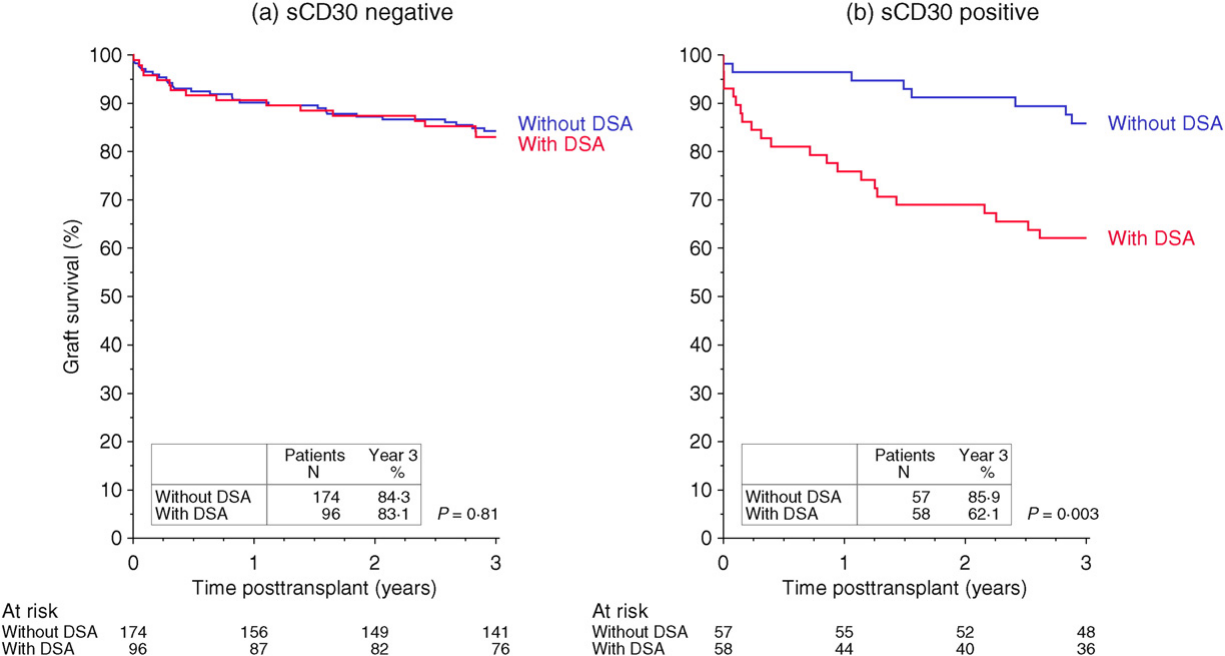
Impact of pretransplant DSA on graft survival. Patients with and without DSA show similar survival rates in the absence of high pretransplant sCD30 (a). In contrast, graft survival is significantly impaired in DSA positive patients if they simultaneously have high pretransplant sCD30 (b).

Our further analysis focused on the 154 patients who possessed SAB-detected pretransplant DSA. As shown in Fig. 1, a deleterious influence of pretransplant DSA on graft survival was evident only in patients who were positive pretransplant for the immune activation marker sCD30. In sCD30 negative patients, 3-year graft survival was nearly identical in patients regardless of the DSA status (sCD30 negative, with DSA: 83.1 ± 3.9% versus sCD30 negative, without DSA: 84.3 ± 2.8%, *P* = 0.81, Fig. 1a). Of all possible combinations of sCD30 and DSA status, the lowest 3-year graft survival was found in the sCD30 positive, with DSA cohort (62.1 ± 6.4%) (Fig. 1b) and was significantly lower than in all the other groups (sCD30 positive, with DSA *P* = 0.003, sCD30 negative, with DSA *P* = 0.003, sCD30 negative, without DSA *P* < 0.001). If the recipients were sCD30 negative, even in the presence of strong DSA reacting with MFI of ≥ 5000 (n = 55) the 3-year graft survival rate was a high 92.6 ± 3.6%, not inferior to the 84.3 ± 2.8% rate in the 174 patients without DSA (*P* = 0.13, data not shown).

When patients who died with a functioning graft were censored, death-censored graft survival rates were equivalent in DSA positive and DSA negative presensitized patients if they were negative for the immune activation marker sCD30 (sCD30 negative, DSA positive vs. sCD30 negative, DSA negative; 86.8 ± 3.6% vs. 89.9 ± 2.3%, respectively, *P* = 0.50, Supplementary Fig. S1a). Only if sCD30 was positive, death censored graft survival was significantly lower in 58 patients who were positive for DSA (74.8 ± 5.9%) than in the 57 presensitized patients who were DSA negative (89.2 ± 4.2%, *P* = 0.036, Supplementary Fig. S1b). In sCD30 positive patients DSA positivity had a significant impact also on patient survival (with DSA 83.3 ± 5.1% vs. without DSA: 96.5 ± 2.4%, *P* = 0.020; Supplementary Fig. S2b).

**Fig. 2:**
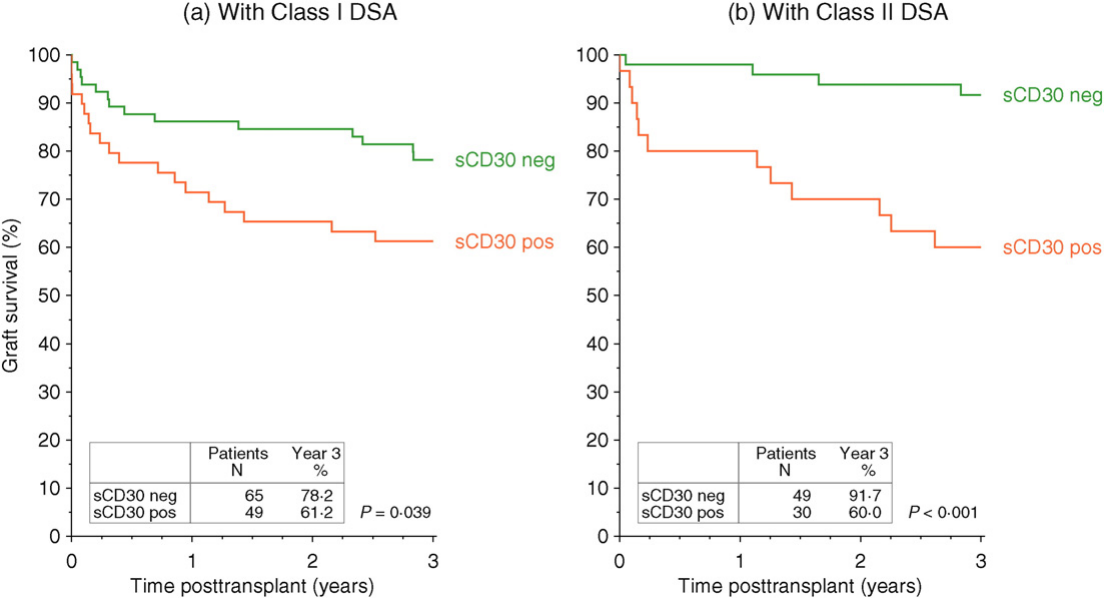
Impact of pretransplant sCD30 on graft survival in patients with class I (a) or class II DSA (b).

Supportive data were obtained when class I or class II DSA positive patients were analyzed separately (Fig. 2). Graft survival was low in class I or class II DSA positive patients who were sCD30 positive (class I DSA: 61.2 ± 7.0%; class II DSA: 60.0 ± 8.9%), significantly inferior to the respective 78.2 ± 5.2% and 91.7 ± 4.0% rates in class I or class II DSA positive patients who were sCD30 negative: *P* = 0.039 and *P* < 0.001, respectively). Even in the co-presence of class I and class II DSA, sCD30 negative patients (n = 18) showed a good 3-year graft survival rate of 88.9 ± 7.4%, as compared to 57.1 ± 10.8% in 21 sCD30 positive patients with class I and class II DSA (*P* = 0.029, Supplementary Fig. S3).

Cox multivariable analysis considering the confounders listed under ‘Methods’ confirmed that the risk of graft loss during the first 3 posttransplant years was not increased in DSA positive patients if they were negative for sCD30 (HR 1.16, 95% CI 0.62–2.19; *P* = 0.64). In contrast, patients positive for DSA as well as sCD30 showed a 2.92 times increased risk of graft loss (HR 2.92, 95% CI 1.60–5.33; *P* < 0.001). The risk was not increased in presensitized patients who were positive only for sCD30 and DSA negative (HR 0.98, 95% CI 0.44–2.21; *P* = 0.97).

## 4 Discussion

This analysis was based on a retrospective study of presensitized patients transplanted between 1996 and 2011, which has the advantage that it was unknown at the time of transplantation whether the patients possessed DSA or not and the recipients were therefore not subjected to special antibody removal desensitization protocols. Because frozen pretransplant patient sera as well as patient and donor DNA samples were available, high resolution HLA typing, high sensitivity SAB antibody specification, as well as serum sCD30 testing could be carried out retrospectively on transplants with already known clinical outcome. Our key finding was that pretransplant DSA had a deleterious impact on graft survival only in the presence of high pretransplant serum levels of the immune activation marker sCD30.

In spite of improvements in kidney graft survival during the last decades, recipients with preformed DSA against donor HLA continue to be at high risk of rejection. Waiting-list patients with broadly-reactive HLA antibodies, by definition, possess DSA against many potential donors and, as a consequence, often experience prolonged waiting times for a deceased donor kidney. Not uncommonly such patients die on the waiting list before they can be transplanted. Live donor transplantation is often denied if potential recipients possess DSA. Some transplant centers perform transplantations after the elimination of DSA by plasma exchange or immunoadsorption, combined with potent immunosuppression which often includes the B- and T-cell-eliminating agents rituximab and ATG, with variable success (Morath et al., 2010, Vo et al., 2015). Interestingly, however, experience has shown that even without special treatment measures, certain patients who possess DSA do well with their transplant (van den Berg-Loonen et al., 2008, Knight et al., 2013, Loupy et al., 2013, Schaefer et al., 2016). These patients should not be exposed unnecessarily to the deleterious side effects of intensified treatment or excluded from transplantation altogether. The practice of enrolling all patients with pretransplant DSA in intensified treatment protocols is defensible only in the absence of a diagnostic tool that allows the differentiation of harmful from non-harmful DSA. Data obtained in the present study indicate that pretransplant determination of the activation marker sCD30 (Susal et al., 2002, Saini et al., 2008) is a diagnostic tool which can differentiate between patients with harmful and harmless DSA.

Perhaps the most important aspect of the current findings is that only 38% of the DSA positive patients were sCD30 positive and thereby identified as truly high risks. As many as 62% of DSA positive patients had a low serum sCD30, suggesting that special treatment measures were not necessary. This applied to patients with DSA against HLA class I, class II, or both, and irrespective of whether the DSA were strong, as indicated by high MFI. We and others have shown previously that the C1q-binding capacity of DSA can serve as a discriminator between deleterious and non-harmful HLA antibodies that appear in the recipient's serum after transplantation (Susal et al., 2015, Loupy et al., 2013, Yabu et al., 2011). In clinical practice, only few patients who possess C1q-binding DSA prior to transplantation are transplanted due to antibody reactivity in the CDC crossmatch. This is a logical explanation why an association of C1q-binding DSA with graft failure could not be established in studies of pretransplant sera (Loupy et al., 2013, Otten et al., 2012). In the present study, pretransplant C1q-binding DSA were detected in only 5 of the 58 sCD30 and DSA positive high-risk patients, an insufficient number for statistically meaningful analysis. Another relevant observation is that, in previous studies, many patients with pretransplant C1q-reactive antibodies appeared to lose their antibody reactivity posttransplant with no apparent deleterious effect on the graft (Susal et al., 2015, Schaefer et al., 2016). In the current study, pretransplant DSA positive, sCD30 negative patients showed virtually identical graft survival rates as DSA negative patients. One would have thought that the presence of DSA at the time of transplantation would be harmful to the graft in any case, if not via complement-activation then through antibody-dependent cytotoxicity which can inflict tissue damage by activating macrophages and natural killer cells. Such was not the case.

Elevated sCD30 may indicate an increased immunological capacity to react against foreign HLA antigens. Due to contact with bioincompatible dialysis membranes and exposure to HLA or HLA-cross-reactive infectious agents (D'Orsogna et al., 2011), end-stage renal disease patients exhibit alterations in their immune response, including increased numbers of interferon (INF)-γ-producing HLA-cross-reactive memory *T*-cells, reduced numbers of regulatory *T*-cells, augmented in vitro expansion of *T*-cells expressing CD30, and an increased serum sCD30 content (Susal et al., 2002, Velásquez et al., 2012). Our group reported that cytomegalovirus infection can lead to an increase of serum sCD30 levels in transplant patients (Weimer et al., 2006). Chan et al. identified CD30 + *T*-cells as the major INF-γ and IL-5 cytokine-producing human *T*-lymphocyte subset generated in response to stimulation with alloantigens (Chan et al., 2002). We showed previously that polyclonal and allogeneic stimulation results in upregulation of CD30 on memory T-cells and an INF-γ-dependent release of sCD30 from these cells (Velasquez et al., 2013). Although a biological function of sCD30 has not been established in vivo, sCD30 has greater affinity for CD30-ligand in vitro as compared to CD30 and is potentially capable of blocking CD30, CD30-ligand interactions (Hargreaves and Al-Shamkhani, 2002), reducing the availability of CD30-ligand on lymphocytes or decreasing the ability of regulatory T-cells to inhibit graft-reactive T-cells (Tarkowski, 2003). Neutralization of IFN-γ resulted in abrogation of sCD30 release from memory T-cells in vitro (Velasquez et al., 2013). High production of IFN-γ by alloreactive anti-donor effector memory T-cells in patients awaiting kidney transplantation has been associated with early acute rejection of kidney transplants (Heeger et al., 1999). The presence of a low-level or declining memory *T*-cell response might serve as an explanation why, despite the presence of DSA, many patients possess low sCD30 levels. Routinely applied immunosuppression appears to be able to control such low-level T-cell-mediated antibody responses.

In sCD30 and DSA positive patients, some 40% of transplanted kidneys failed within 3 years. This rate is unacceptable. Allocation of HLA well-matched donor kidneys to these truly “high risk” patients may be an effective approach to minimize graft loss. It was shown in previous studies that deleterious sCD30 as well as HLA antibody effects can successfully be compensated by good HLA matching (Susal et al., 2003, Susal et al., 2009). Special desensitization and immunosuppression programs may be suitable alternatives. Because of the side effects associated with such treatment, many centers do not perform transplants from live donors if DSA are present. Our data suggest that transplantations can be safely performed in DSA positive patients if the pretransplant sCD30 level is low. However, before such policy can be generally implemented, further studies are required that confirm the present findings.

Limitations of the present study are its retrospective nature and the lacking demonstration of the mechanism how DSA exert their harmful effect in dependence of a preactivated immune system. Switch of B cells under T-cell support to long-lived plasma cells, which produce harmful antibody isotypes, and a potential predisposition of certain patients to the simultaneous production of sCD30 and persistent DSA are issues that remain to be addressed in future studies. Strengths of the study are the robustness of the presented findings, which due to the strong impact on graft outcome should be readily reproducible by other research groups, and their technically uncomplicated potential applicability for clinical routine.

In conclusion, our data indicate that the presence of pretransplant DSA is associated with a high rate of graft rejection only if patients concomitantly have high levels of pretransplant sCD30 which reflect a preactivated immune response. For practical purposes, it appears that only patients positive for both DSA and sCD30 require special therapeutic and organizational measures, such as the elimination of DSA from the patient's circulation, potent immunosuppression, good HLA matching and intense posttransplant monitoring (Morath et al., 2010, Susal et al., 2003, Susal et al., 2009). According to this analysis, the majority of pretransplant DSA positive patients exhibit low sCD30 levels and can be expected to enjoy good graft survival without special provisions.

## Contributors

CS, BD, and GO designed the study. AR and BD selected the patients from the database. CS, SS, THT, and PG supervised the testings. BD performed the statistical analyses. CS and GO wrote the manuscript. AS, TF, EW, BK, MR, SB, SZC, DJM, DK, MPE, PP, CB, RW, JM, PS, MZ collected and provided sera, DNA and clinical data. CM, EW, SZC, DJM, RW critically reviewed the manuscript. All authors checked and approved the final manuscript.

## Declaration of Interests

We declare no competing interests.

## Figures and Tables

**Table 1:** Demographics of study patients⁎.

Characteristic	Without DSA n = 231	With DSA n = 154	Total n = 385
Geographical region Europe North America	175 (76%) 56 (24%)	138 (90%) 16 (10%)	313 (81%) 72 (19%)
Transplant year 1996–2001 2002–2006 2007–2011	39 (17%) 88 (38%) 104 (45%)	25 (16%) 61 (40%) 68 (44%)	64 (17%) 149 (39%) 172 (45%)
Transplant number First transplant Retransplant	159 (69%) 72 (31%)	67 (44%) 87 (56%)	226 (59%) 159 (41%)
Recipient sex Female Male	104 (45%) 127 (55%)	90 (58%) 64 (42%)	194 (50%) 191 (50%)
Recipient race^a^ Caucasian Other	203 (99%) 3 (1%)	135 (98%) 3 (2%)	338 (98%) 6 (2%)
Recipient age (years) Mean ± SD	49.3 ± 13.0	49.7 ± 12.1	49.4 ± 12.6
Donor age (years) Mean ± SD	47.6 ± 16.7	47.2 ± 17.5	47.5 ± 17.0
HLA-A + B + DR mismatches 0–1 2–4 5–6	65 (28%) 146 (63%) 20 (9%)	23 (15%) 112 (73%) 19 (12%)	88 (23%) 258 (67%) 39 (10%)
Initial immunosuppression^b^ CNI Mycophenolates Steroids	221 (97%) 210 (92%) 220 (96%)	143 (94%) 144 (95%) 150 (99%)	364 (96%) 354 (93%) 370 (97%)
Antibody induction therapy^b^ ATG IL2-RA Other None	16 (7%) 85 (37%) 4 (2%) 123 (54%)	27 (18%) 45 (30%) 1 (1%) 79 (52%)	43 (11%) 130 (34%) 5 (1%) 202 (53%)
IgG-anti-HLA antibodies Class I positive Class II positive	89 (39%) 69 (30%)	96 (62%) 98 (64%)	185 (48%) 167 (43%)
Soluble CD30 (ng/ml) < 80 ≥ 80	174 (75%) 57 (25%)	96 (62%) 58 (38%)	270 (70%) 115 (30%)
3-year follow-up Complete Incomplete	224 (97%) 7 (3%)	150 (97%) 4 (3%)	374 (97%) 11 (3%)

SD, standard deviation; CNI, calcineurin inhibitors; ATG, antithymocyte globulin; IL2-RA, interleukin-2 receptor antagonist.

⁎ There are significant differences between patients with and without DSA in geographical region (*P* < 0.001), transplant number (*P* < 0.001), recipient sex (*P* = 0.012), HLA-A + B + DR mismatches (*P* = 0.007), antibody induction therapy (*P* = 0.008), IgG-anti-HLA antibodies (*P* < 0.001) and soluble CD30 (*P* = 0.006).

a Data on race are not collected at transplant centers in some European countries due to legal restriction.

b Immunosuppressive medication of three patients without DSA and two patients with DSA was unknown.

## Appendix A Supplementary data

Supplementary material. Image 1
