# Supplementary material for: Donor-specific antibodies require preactivated immune system to harm renal transplant
Source: eBioMedicine. 2016 Jun 5;9:366–71. doi: 10.1016/j.ebiom.2016.06.006 (PMC4972543; doi:10.1016/j.ebiom.2016.06.006)
Supplement: Supplementary file 1 — Supplementary material. [file mmc1.pdf]

Supplementary Materials

Donor-specific antibodies require preactivated immune system to harm renal transplant

Caner Süsal[1]*, Bernd Döhler[1], Andrea Ruhenstroth[1], Christian Morath[2], Antonij Slavcev[3], Thomas Fehr[4], Eric Wagner[5], Bernd Krüger[6], Margaret Rees[7], Sanja Balen[8], Stela Živčić-Ćosić[8], Douglas J. Norman[9], Dirk Kuypers[10], Marie-Paule Emonds[11], Przemyslaw Pisarski[12], Claudia Bösmüller[13], Rolf Weimer[14], Joannis Mytilineos[15] Sabine Scherer[1], Thuong H. Tran[1], Petra Gombos[1], Peter Schemmer[16], Martin Zeier[2], and Gerhard Opelz[1]

A Collaborative Transplant Study Report

[1]Transplantation Immunology, Institute of Immunology and [2]Division of Nephrology, University of Heidelberg, Heidelberg, Germany; [3]Department of Immunogenetics, Institute for Clinical and Experimental Medicine (IKEM), Prague, Czech Republic; [4]Division of Nephrology, University Hospital Zurich, Zurich, Switzerland; [5]Immunology and Histocompatibility Laboratory, CHU de Québec-Université Laval, and Department of Microbiology, Infectious Diseases and Immunology, Université Laval, Québec, QC, Canada; [6]University Medical Centre Mannheim, Mannheim, Germany; [7]Welsh Transplantation and Immunogenetics Laboratory; Cardiff, United Kingdom; [8]Departments of Transfusion Medicine and Nephrology, Dialysis, Kidney Transplantation, University Hospital Centre, Rijeka, Croatia; [9]Oregon Health and Science University, Portland, OR, USA; [10]Department of Nephrology and Renal Transplantation and [11]Blood Transfusion Center, University Hospitals Leuven, Leuven, Belgium; [12]Transplantation Surgery, University of Freiburg, Freiburg, Germany; [13]Department of General and Transplant Surgery, Innsbruck Medical University, Innsbruck, Austria; [14]Department of Internal Medicine, University Clinic of Giessen and Marburg, Giessen, Germany; [15]Department of Transplantation Immunology, Institute of Transfusion Medicine, University Clinic Ulm, Ulm, Germany; [16]Transplantation and General Surgery, University of Heidelberg, Heidelberg, Germany

Supplementary Table S1. Demographics of study patients*

Characteristic	Without DSA		With DSA	
	sCD30 neg n=174	sCD30 pos n=57	sCD30 neg n=96	sCD30 pos n=58
Geographical region				
Europe	131 (75%)	44 (77%)	84 (88%)	54 (93%)
North America	43 (25%)	13 (23%)	12 (12%)	4 (7%)
Transplant year				
1996 – 2001	30 (17%)	9 (16%)	15 (16%)	10 (17%)
2002 – 2006	60 (34%)	28 (49%)	34 (35%)	27 (47%)
2007 – 2011	84 (48%)	20 (35%)	47 (49%)	21 (36%)
Transplant number				
First transplant	128 (74%)	31 (54%)	38 (40%)	29 (50%)
Retransplant	46 (26%)	26 (46%)	58 (60%)	29 (50%)
Recipient sex				
Female	75 (43%)	29 (51%)	54 (56%)	36 (62%)
Male	99 (57%)	28 (49%)	42 (44%)	22 (38%)
Recipient race**				
Caucasian	152 (99%)	51 (98%)	83 (98%)	52 (98%)
Other	2 (1%)	1 (2%)	2 (2%)	1 (2%)
Recipient age (years)				
Mean ± SD	50·7 ± 12·2	45·0 ± 14·4	50·8 ± 10·7	47·8 ± 13·8
Donor age (years)				
Mean ± SD	48·9 ± 16·0	43·7 ± 18·2	47·4 ± 16·8	46·9 ± 18·7
HLA-A+B+DR mismatches				
0 – 1	48 (28%)	17 (30%)	15 (16%)	8 (14%)
2 – 4	112 (64%)	34 (60%)	69 (72%)	43 (74%)
5 – 6	14 (8%)	6 (11%)	12 (12%)	7 (12%)
Initial immunosuppression†				
CNI	166 (97%)	55 (98%)	89 (94%)	54 (95%)
Mycophenolates	156 (91%)	54 (96%)	89 (94%)	55 (96%)
Steroids	166 (97%)	54 (96%)	94 (99%)	56 (98%)
Antibody induction therapy†				
ATG	11 (6%)	5 (9%)	19 (20%)	8 (14%)
IL2-RA	63 (37%)	22 (39%)	27 (28%)	18 (32%)
Other	3 (2%)	1 (2%)	1 (1%)	0 (0%)
None	95 (55%)	28 (50%)	48 (51%)	31 (54%)
IgG-anti-HLA antibodies				
Class I positive	63 (36%)	26 (46%)	52 (54%)	44 (76%)
Class II positive	52 (30%)	17 (30%)	64 (67%)	34 (59%)
3-years follow up				
Complete	168 (97%)	56 (98%)	92 (96%)	58 (100%)
Incomplete	6 (3%)	1 (2%)	4 (4%)	0 (0%)

* There are a significant differences for patients ‘Without DSA’: transplant number (*P*=0·007), recipient age (*P*=0·008), donor age (*P*=0·050); patients ‘With DSA’: IgG-anti-HLA Class I antibodies (*P*=0·007).

** Data on race are not collected at transplant centres in some European countries due to legal restriction.

† immunosuppressive medication of three patients without DSA and two patients with DSA is unknown.

SD, standard deviation; CNI, Calcineurin inhibitors; ATG, antithymocyte globulin; IL2-RA, interleukin-2 receptor antagonist.

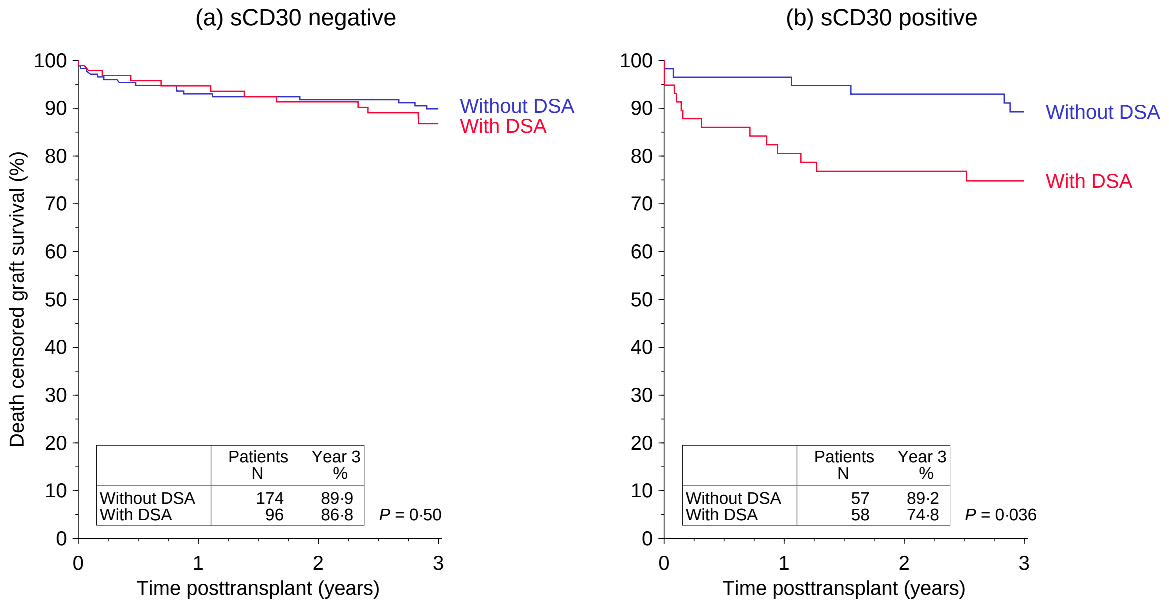

Supplementary Figure S1. Impact of pretransplant DSA on death censored graft survival in a) sCD30 negative and b) sCD30 positive patients.

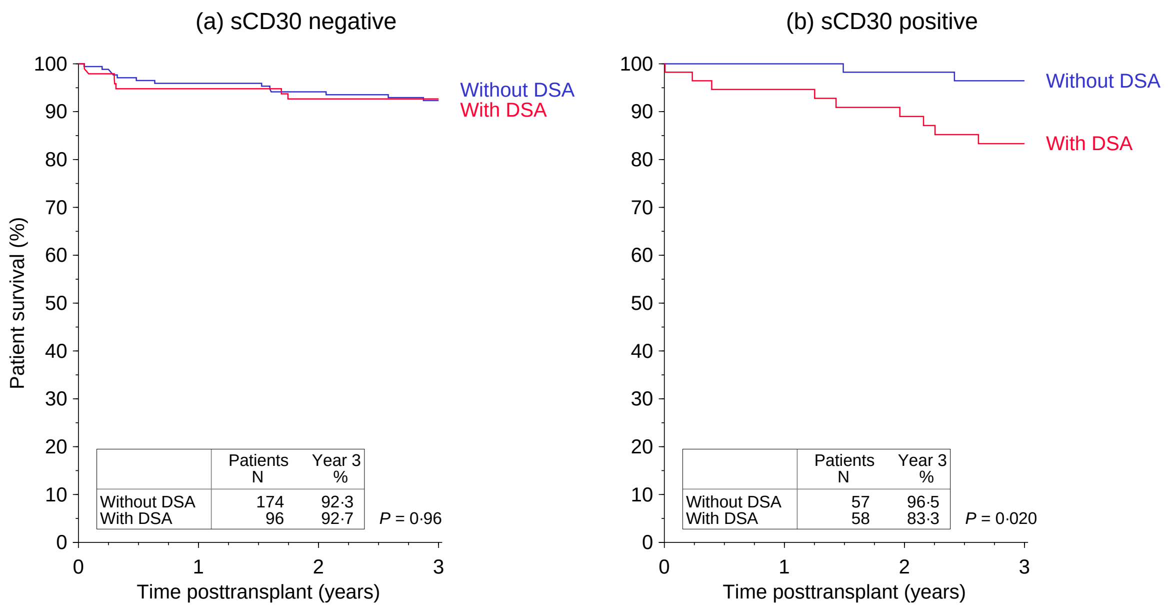

Supplementary Figure S2. Impact of pretransplant DSA on patient survival in a) sCD30 negative and b) sCD30 positive patients.

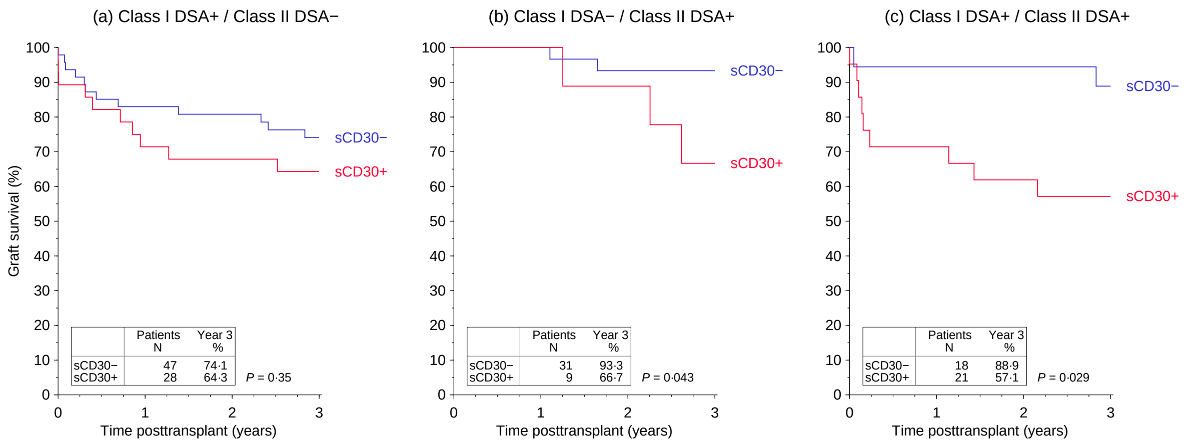

Supplementary Figure S3. Impact of pretransplant sCD30 on graft survival in patients with class I and/or class II DSA positivity.
